# Reduced lung function during childhood in identical twins with discordant fetal growth: a cohort study

**DOI:** 10.1016/j.eclinm.2024.102600

**Published:** 2024-04-10

**Authors:** Jip A. Spekman, Joël Israëls, Ilja de Vreede, Mady Los, Miranda J.J. Geelhoed, Erik W. van Zwet, Monique C. Haak, Arno A.W. Roest, Jeanine M.M. van Klink, Enrico Lopriore, Sophie G. Groene

**Affiliations:** a Neonatology, Department of Pediatrics, Willem-Alexander Children's Hospital, Leiden University Medical Centre, Leiden, the Netherlands; b Pediatric Pulmonology, Department of Pediatrics, Willem-Alexander Children's Hospital, Leiden University Medical Centre, Leiden, the Netherlands; c Department of Pulmonology, Leiden University Medical Centre, Leiden, the Netherlands; d Medical Statistics, Department of Biomedical Data Sciences, Leiden University Medical Centre, Leiden, the Netherlands; e Fetal Therapy, Department of Obstetrics, Leiden University Medical Centre, Leiden, the Netherlands; f Pediatric Cardiology, Department of Pediatrics, Willem-Alexander Children's Hospital, Leiden University Medical Centre, Leiden, the Netherlands

**Keywords:** Monochorionic twins, Selective fetal growth restriction, Lung function, Spirometry

## Abstract

**Background:**

Fetal growth restriction (FGR) can negatively affect lung development, leading to increased respiratory morbidity and reduced lung function later in life. Studies regarding the impact of FGR on lung function in singletons are influenced by genetic, obstetric, and maternal factors. To overcome these confounding factors, we aim to investigate lung function in identical twins with selective FGR (sFGR).

**Methods:**

Lung function assessments were performed in identical twins with sFGR born in our centre between March 1, 2002, and December 31, 2017, aged between 5 and 17 years. sFGR was defined as birthweight discordance ≥20%. Outcome measures consisted of forced expiratory volume in 1 s (FEV_1_), forced vital capacity (FVC), and transfer factor for carbon monoxide (DLCO) and were compared between the smaller and larger twin.

**Findings:**

Thirty-nine twin pairs performed spirometry of sufficient quality. Median gestational age at birth was 34.3 (interquartile range (IQR) 32.1–36.0) weeks with median birthweights of 1500 (IQR 1160–1880) grams and 2178 (IQR 1675–2720) grams for the smaller and larger twin, respectively. Smaller twins had significantly lower z-scores for FEV_1_ (−0.94 versus −0.41, *p* = 0.0015), FVC (−0.56 versus −0.06, *p* < 0.0001) and DLCO (−0.50 versus 0.00, *p* < 0.0001) compared to larger co-twins.

**Interpretation:**

Although being genetically identical, sFGR in identical twins is associated with a reduction in static and dynamic lung volume and a reduction in lung diffusion, even when taking the reduced lung volume into account. This indicates that adverse growth conditions in utero negatively affect lung development and function, potentially contributing to an increase in respiratory morbidities later in life.

**Funding:**

The 10.13039/100002129Dutch Heart Foundation and The 10.13039/501100005040Bontius Foundation.

**Research in context**

**Evidence before this study**

We searched PubMed on January 4, 2024, with the search terms “fetal growth restriction” and “lung function” without date or language restrictions. Available literature showed that fetal growth restriction (FGR) is associated with reduced lung function during childhood, albeit primarily based on studies in which singletons with FGR are compared to appropriately-grown singletons. These studies are often susceptible to confounding factors including genetic, obstetric, parental, and postnatal environmental influences, which can contribute to a reduction in lung function as well.

**Added value of this study**

We present comprehensive lung function assessments, including spirometry, diffusion capacity of the lung for carbon monoxide, and multiple-breath helium dilution method in a large cohort of identical twins with discordant fetal growth. These twins are a unique study population in which many potential confounders of childhood lung function are naturally eliminated.

**Implications of all the available evidence**

This study suggests that FGR has a persistent negative effect on lung function during childhood, independent from confounding factors. Although the lung function deficit was modest in most children, having reduced lung volumes and diffusion capacity in the smaller twin can contribute to an increase in respiratory morbidities later in life. Therefore, screening in those with concomitant risk factors and timely counselling of additional hits on lung function is warranted in those born after FGR. These findings can serve as basis to further investigate the achievement of maximum lung capacity after FGR in discordant identical twins.

## Introduction

Fetal growth restriction (FGR) due to prolonged deprivation of nutrients and oxygen in utero is thought to negatively affect the development of multiple organs, including the lungs.^1^ Lung development is a complex process that begins in early fetal life and continues until early adulthood.^2^^,^^3^ Impaired lung development can result in reduced lung function, subsequently increasing susceptibility to respiratory morbidities during childhood and in later life.

Increasing evidence shows that FGR is associated with reduced lung function.^2^^,^^4^, ^5^, ^6^ Specifically, multiple studies revealed a reduction in forced expiratory volume in 1 s (FEV_1_) and forced vital capacity (FVC), whereas the association between FGR and FEV_1_/FVC ratio varies among studies. Reduced lung function in low birthweight children can lead to asthma like symptoms but are rather attributed to prematurity.^5^ However, disentangling the effects of FGR and prematurity is challenging as they both affect lung function but through different mechanisms, thereby making it difficult to attribute the observed effects to either factor.^3^, ^4^, ^5^ Additionally, clinical studies are often susceptible to confounding factors including genetic, obstetric, maternal, and postnatal environmental influences, which can also reduce lung function. A unique study population in which many potential confounders are naturally eliminated are monochorionic (MC) twins.

MC twins are monozygotic (i.e. genetically identical) twins who share a single placenta. Approximately 10–15% of MC twin pregnancies present with an unequal sharing of the placenta, resulting in an imbalanced supply of oxygen and nutrients, subsequently leading to a large growth discordance within twin pairs.^7^ This large growth discordance is termed selective fetal growth restriction (sFGR) when the intertwin weight difference is ≥20%. Thus, in twin pairs affected by sFGR, a comparison can be made between a smaller, growth-restricted twin and its genetically identical appropriately-grown co-twin born at the same gestational age. At present, data on the long-term effects of sFGR on lung function is scarce. The few available twin studies did not differentiate in chorionicity, lacked comprehensive outcome measures of lung function, and comprised a small sample size.^8^, ^9^, ^10^, ^11^ This study aims to investigate the effect of FGR on lung function during childhood by conducting a within-pair analysis between the smaller and larger twin in MC twin pairs with sFGR, naturally correcting for potential confounding factors influencing lung development.

## Methods

### Study design

This study is part of The LEMON study (Long-term Effects of selective fetal growth restriction in MONochorioc twins, International Clinical Trial Registry Platform ID NL9833), a cohort study conducted at the Leiden University Medical Centre (LUMC), which serves as the expertise centre for complicated MC twin pregnancies and fetal therapy in The Netherlands.^12^ The LEMON study aimed to investigate the long-term effects of sFGR in MC twins. It was reviewed and approved by the ethics committee of the LUMC (P20.089). Informed consent was obtained from parents and/or children ≥12 years of age, and inclusion was completed on January 31, 2022. Neurodevelopmental, psychosocial and growth pattern outcomes from the LEMON study were previously published.^12^, ^13^, ^14^

All MC twins diagnosed with sFGR residing in the Netherlands and born between March 1, 2002, and December 31, 2017, were eligible for inclusion, utilizing a convenience sampling method. sFGR was defined as birthweight discordance (BWD) ≥20%, which was calculated using the formula (birthweight of the larger twin  – birthweight of the smaller twin)/birthweight of the larger twin × 100. This definition serves as a postnatal expression of discordant antenatal growth. We excluded twin pregnancies diagnosed with monoamnionicity, twin–twin transfusion syndrome (TTTS),^15^ twin anemia polycythemia sequence (TAPS),^16^ twin reversed arterial perfusion,^17^ or other congenital abnormalities. In addition, to enable within-pair analysis, we excluded twin pairs in which one or both twins died.

The following maternal and perinatal baseline characteristics were retrospectively collected: maternal age at delivery, gravidity, parity, maternal smoking, maternal hypertensive disease (i.e. gestational hypertension, defined as a rise in systolic blood pressure ≥140 mmHg and/or diastolic blood pressure ≥90 mmHg, or preeclampsia, defined as hypertension accompanied by proteinuria ≥300 mg/24 h), administration of a full course of corticosteroids prior to delivery, type of sFGR defined according to the Gratacós type classification,^18^ gestational age at birth, sex, delivery mode, BWD, birthweight, small for gestational age (SGA; i.e. birthweight <10th centile),^19^ placental share (i.e. expressed as percentage of the total placental area, based on twin-specific dye margins after standard colour dye injection of MC twin placentas), severe neonatal morbidity, respiratory distress syndrome (RDS; i.e. respiratory failure requiring mechanical ventilation and/or surfactant),^20^ bronchopulmonary dysplasia (BPD; defined as supplemental oxygen for ≥28 days),^21^ need for surfactant, proportion of twins who received mechanical ventilation and non-invasive respiratory support (defined as continuous positive airway pressure, high flow and low flow). Severe neonatal morbidity was defined as a composite outcome of at least one of the following: RDS; persistent pulmonary hypertension of the neonate (i.e. failure of circulatory transition after birth requiring treatment with nitric oxide); patent ductus arteriosus requiring medical treatment or surgical closure; necrotising enterocolitis of at least stage 2; neonatal sepsis (i.e. a clinically ill neonate with positive blood cultures); BPD; and severe cerebral injury (i.e. intraventricular haemorrhage ≥ grade 3, cystic periventricular leukomalacia ≥ grade 2, ventricular dilatation >97th percentile, arterial or venous infarction, or porencephalic or parenchymal cysts).^12^ Childhood characteristics consisted of reported use of asthma medication (e.g. albuterol, fluticasone, beclomethasone), age at spirometry and height. Data on maternal smoking and use of asthma medication were obtained using a standardized parental questionnaire. Height of the children was measured at the time of the spirometry.

Between March 2021 and April 2023, a follow-up appointment was arranged at the outpatient clinic of the LUMC to perform comprehensive lung function tests according to the American Thoracic Society and European Respiratory Society Guideline and deemed feasible in preschool-aged children.^22^ The children underwent simultaneous lung function assessments with a duration of approximately 45 min per child under the supervision of a respiratory physiologist, during which no bronchodilators were administered. Data were used for analysis when both children of a twin pair demonstrated technically acceptable and reproducible measurements of that outcome, reviewed by a paediatric pulmonologist when necessary.

Lung function assessments included spirometry, diffusion capacity of the lung for carbon monoxide (DLCO) using the single-breath CO-diffusion method, and multiple-breath helium dilution method. Not all twin pairs underwent the last two tests, as these tests were added to the study in August 2021 (start of study in March 2021). Lung function measures comprised forced expiratory volume in 1 s (FEV_1_), forced vital capacity (FVC), FEV_1_/FVC ratio, maximum vital capacity (VC MAX), FEV_1_/VC MAX ratio, DLCO (i.e. the ability of the lungs to transfer inhaled gas to haemoglobin in pulmonary capillary blood), alveolar volume (VA), carbon monoxide transfer coefficient (KCO; diffusion capacity related to VA), total lung capacity (TLC), residual volume (RV) and RV/TLC ratio. Absolute values of all measurements were converted into age-, height-, sex- and ethnicity-adjusted z-scores following the Global Lung Function Initiative reference values, eliminating confounding of these factors.^23^ The mean within-pair difference for all outcome measurements was calculated as outcome larger twin—outcome smaller twin. The lower limit of normal (LLN) was defined as a z-score less than −1.64.^24^

### Statistical analysis

Statistical analyses were performed using IBM SPSS Statistics for Windows version 29.0 (IBM Corp., Armonk, NY, USA) and R version 4.1.2 (R Foundation for Statistical Computing, Vienna, Austria). Data are presented as median (interquartile range (IQR)), mean (standard deviation (SD)) or n/N (%). Within-pair differences in lung function parameters between the smaller and larger twin were analysed using a paired t-test, as the normality of differences assumption was met. This analysis considers the lack of independence between observations of co-twins. We exclusively tested z-scores as these are age-, height-, sex- and ethnicity-adjusted and thereby eliminate confounding of these respective factors, allowing us to focus on the true effect of (s)FGR on lung function. Risk ratios for having a z-score below the LLN were calculated using a generalised estimating equation with a Poisson regression model, an unstructured correlation matrix, and cluster-robust standard errors. In addition, a subgroup analysis was performed excluding twin pairs in which one or both twins had BPD to investigate whether the observed differences in lung function are attributable to BPD sequelae rather than FGR. Furthermore, a univariate linear regression analysis was used to evaluate the impact of age at spirometry on within-pair difference in FEV_1_ and FVC z-scores, providing insights into the increase or decrease in lung function disparities over time during childhood in these twins. The analysis was performed under the assumptions of linearity, normality and homoscedasticity, and no time-varying confounding. A *p*-value <0.05 was considered to indicate statistical significance.

### Role of the funding source

The funding source had no role in study design, data collection, data analysis, data interpretation, or writing of the report. All authors had full access to all the data in the study and had final responsibility for the decision to submit for publication.

## Results

**Fig. 1:**
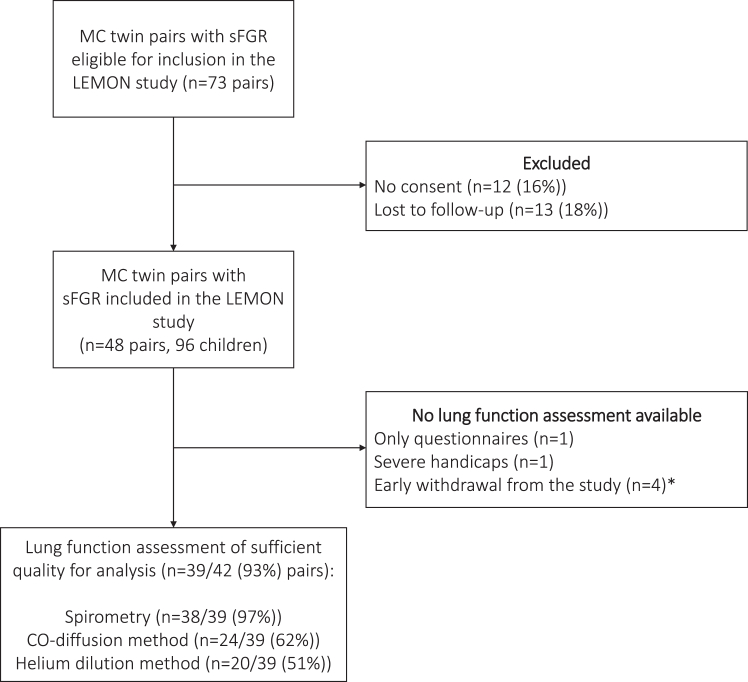
Flowchart of LEMON study inclusion. MC: monochorionic, sFGR: selective fetal growth restriction, CO: carbon monoxide. ∗Early withdrawal due to hospital anxiety or for logistical reasons.

Between 2002 and 2017, 73 twin pairs were eligible for inclusion in the LEMON study. Of these twin pairs, 12 (16%) did not give consent for participating in the LEMON study and 13 (18%) were lost to follow-up (5 were not residing in the Netherlands anymore and 8 could not be reached), leaving 48 twin pairs to be included in the LEMON study (Fig. 1). Baseline characteristics were compared between the twin pairs who were included in the LEMON study and twin pairs who did not give consent or were lost to follow-up (n = 25 (34%)), and no significant differences were identified.^12^ Lung function assessment was unavailable in 6/48 (13%) twin pairs due to participation only in the questionnaire assessment of the study, severe handicaps, and early withdrawal from the study (due to hospital anxiety or for logistical reasons). Ultimately, 42 twin pairs performed spirometry in our outpatient clinic, of which 39/42 (93%) were of sufficient quality. CO-diffusion and helium dilution measurements of sufficient quality were performed in 24/39 (62%) and 20/39 (51%) twin pairs, respectively.

Maternal, obstetrical and perinatal baseline characteristics of the children included are presented in Table 1. Median gestational age at birth was 34.3 (IQR 32.1–36.0) weeks with a median birthweight of 1500 (IQR 1160–1880) grams for the smaller twin and 2178 (IQR 1675–2720) grams for the larger twin. Reported use of asthma medication was 13% (5/39) among smaller twins and 8% (3/39) among larger twins. Mean age at spirometry was 11.6 (SD 3.2) years (Table 2).

**Table 1:** Maternal and perinatal baseline characteristics for MC twin pairs with sFGR.

Characteristics	MC twins (n = 78; 39 pregnancies)	Smaller twin (n = 39)	Larger twin (n = 39)
Maternal age (years)	32 (29–35)	–	–
Gravidity	2 (1–2)	–	–
Parity	0 (0–1)	–	–
Maternal smoking during pregnancy	6/38 (16)^a^	–	–
Maternal hypertensive disease	10/39 (26)	–	–
Full course of corticosteroids prior to delivery	16/33 (48)^b^	–	–
Gratacós type^c^			
Type I	23/39 (59)	–	–
Type II	8/39 (21)	–	–
Type III	8/39 (21)	–	–
Gestational age at birth (weeks)	34.3 (32.1–36.0)	–	–
Female	46/78 (59)	–	–
Caesarean delivery	40/78 (51)	–	–
Birthweight discordance (%)	29.6 (25.1–32.4)	–	–
Birthweight (grams)	–	1500 (1160–1880)	2178 (1675–2720)
Small for gestational age	–	38/39 (97)	8/39 (21)
Placental share (%)^d^	–	31.3 (27.4–41.4)	68.7 (58.6–72.6)
Severe neonatal morbidity	–	7/39 (18)	8/39 (21)
RDS	–	1/39 (3)	6/39 (15)
BPD	–	3/39 (8)	2/39 (5)
Neonatal respiratory outcomes			
Need for surfactant	–	0/39 (0)	5/39 (13)
Mechanical ventilation	–	2/39 (5)	5/39 (13)
Non-invasive respiratory support	–	15/39 (39)	13/39 (33)

Outcomes are presented as median (IQR) or n/N (%). MC: monochorionic, sFGR: selective fetal growth restriction, RDS: respiratory distress syndrome, BPD: bronchopulmonary dysplasia.

a 1 missing value.

b 6 missing values.

c Type I: positive end-diastolic flow, type II: absent or reversed end-diastolic flow, type III: intermittent absent or reversed end-diastolic flow.

d 7 missing values in either group.

**Table 2:** Within-pair comparison of spirometry, CO-diffusion, and helium dilution outcomes between the smaller and larger twin in MC twins with sFGR.

	Smaller twin (n = 39)	Larger twin (n = 39)	*p*-value	Mean difference (95% CI)
Age at follow-up (years)	11.6 (3.2)		–	–
Height at follow-up (cm)	149.3 (20.0)	152.1 (20.1)	<0.0001	2.8 (1.9–3.7)
Spirometry (n = 38)^a^				
FEV_1_ (L)	2.30 (1.01)	2.54 (1.04)	–	0.24 (0.14–0.34)
z-score	−0.94 (1.00)	−0.41 (1.12)	0.0015	0.54 (0.22–0.85)
FVC (L)	2.77 (1.22)	3.05 (1.33)	–	0.28 (0.20–0.37)
z-score	−0.56 (0.84)	−0.06 (1.07)	<0.0001	0.50 (0.28–0.72)
FEV_1_/FVC	83.21 (7.34)	83.92 (6.87)	0.57	0.72 (−1.82–3.26)
VC MAX (L)	2.82 (1.19)	3.10 (1.30)	–	0.28 (0.19–0.37)
z-score	−0.48 (0.95)	−0.04 (1.10)	<0.0001	0.44 (0.25–0.62)
FEV_1_/VC MAX	82.57 (7.96)	83.32 (6.83)	0.58	0.74 (−1.97–3.46)
CO-diffusion method (n = 24)^a^				
DLCO (mmol/(min^a^kPa))	5.34 (1.47)	6.07 (1.68)	–	0.73 (0.45–1.02)
z-score	−0.50 (0.89)	0.00 (0.86)	<0.0001	0.50 (0.25–0.75)
VA (L)	3.09 (0.91)	3.36 (0.99)	–	0.27 (0.14–0.38)
z-score	−0.42 (0.93)	−0.17 (0.93)	0.011	0.25 (0.06–0.43)
KCO (mmol/(min^a^kPa))	1.78 (0.24)	1.86 (0.24)	–	0.08 (0.02–0.15)
z-score	−0.16 (0.70)	0.19 (0.70)	0.0053	0.35 (0.12–0.58)
Helium dilution method (n = 20)				
RV (L)	0.87 (0.28)	0.86 (0.28)	–	0.00 (−0.11–0.10)
z-score	0.12 (0.45)	0.01 (0.51)	0.46	−0.11 (−0.42–0.20)
TLC (L)	3.32 (0.89)	3.56 (0.95)	–	0.24 (0.09–0.38)
z-score	−0.35 (0.97)	−0.15 (0.79)	0.14	0.19 (−0.07–0.45)
% predicted	95.9 (13.9)	98.3 (10.7)	0.18	2.4 (−1.2–6.0)
RV/TLC	26.34 (5.73)	24.49 (7.09)	0.24	−1.84 (−5.01–1.32)

Outcomes are presented as mean (SD) and as mean difference (95% CI). All analysis were performed using the paired t*-*test. The within-pair difference is calculated as: outcome larger twin—outcome smaller twin. Cm: centimetres, FEV_1_: forced expiratory volume in 1 s, L: litres, FVC: forced vital capacity, VC MAX: maximum vital capacity, CO: carbon monoxide, DLCO: diffusion capacity of the lung for carbon monoxide, mmol: millimoles, min: minutes, kPa: kilopascal, VA: alveolar volume, L: litres, KCO: carbon monoxide transfer coefficient, RV: residual volume, L: litres, TLC: total lung capacity.

a Not all spirometry outcomes were of sufficient quality in all of the 39 twin pairs, resulting in outcome measures available in either 37 (FEV_1_/VC MAX) or 38 (all other outcomes) twin pairs. Regarding CO-diffusion method, VA and KCO was available in 23 twin pairs.

**Fig. 2:**
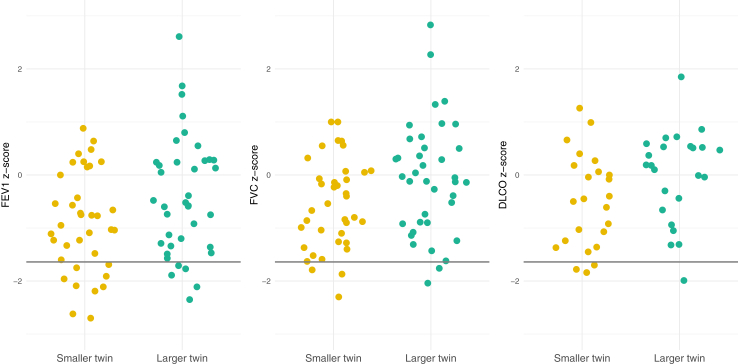
Sinaplot of FEV_1_ z-scores, FVC z-scores and DLCO z-scores for the smaller and larger twin. The width of graph represents the distribution of the data and the grey line represents the lower limit of normal (z-score of −1.64). FEV_1_: forced expiratory volume in 1 s, FVC: forced vital capacity, DLCO: diffusion capacity of the lung for carbon monoxide.

**Fig. 3:**
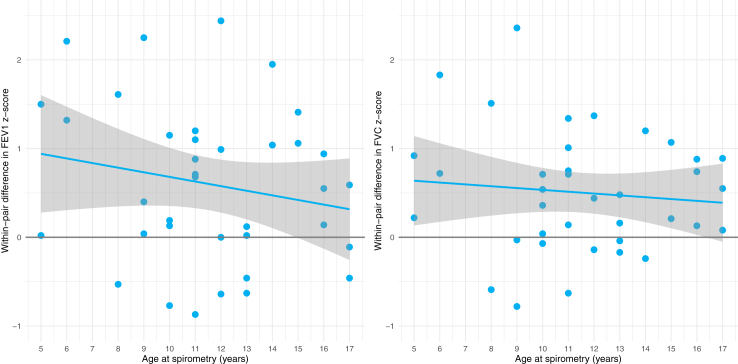
Scatterplot of within-pair differences in FEV_1_ and FVC z-scores against age at spirometry in years. The calculation of the within-pair differences was z-score larger twin—z-score smaller twin. The grey line indicates no z-score difference between the larger and smaller twin. A positive difference (i.e. above the grey line) indicates that the larger twin had a higher z-score than the smaller twin. A negative difference (i.e. below the grey line) indicates that the larger twin had a lower z-score than the smaller twin. The blue lines are the regression lines with corresponding 95% confidence interval for within-pair difference in FEV_1_ z-score (β coefficient −0.042 (95% CI −0.140 to 0.056), *p* = 0.39) and within-pair difference in FVC z-score (β coefficient −0.021 (95% CI −0.090 to 0.048), *p* = 0.55). FEV_1_: forced expiratory volume in 1 s, FVC: forced vital capacity.

We found lower z-scores in the smaller twin compared to the larger twin in all spirometry measurements. FEV_1_ z-scores showed a mean difference of 0.54 (95% CI 0.22–0.85, *p* = 0.0015), and were below the LLN in 10/38 (26%) of the smaller twins and 5/38 (13%) of the larger twins, with a risk ratio of 2.0 (95% CI 1.0–4.2) (Fig. 2). FVC z-scores showed a mean difference of 0.50 (95% CI 0.28–0.72, *p* < 0.0001), and were below the LLN in 3/38 (8%) of the smaller twins and 2/38 (5%) of the larger twins, with a risk ratio of 1.5 (95% CI 0.4–6.0). Additionally, VC MAX z-scores showed a mean difference of 0.28 (95% CI 0.19–0.37, *p* < 0.0001). Univariate linear regression to test the influence of age at spirometry on within-pair difference in FEV_1_ z-score (β coefficient −0.042 (95% CI −0.140 to 0.056), *p* = 0.39) and the within-pair difference in FVC z-score (β coefficient −0.021 (95% CI −0.090 to 0.048), *p* = 0.55) showed no significant increase or decrease over time as depicted in Fig. 3.

In terms of diffusion capacity of the lung for CO, all outcomes showed the same association with lower z-scores in the smaller as opposed to the larger twin. DLCO showed a mean difference of 0.50 z-score (95% CI 0.25–0.75, *p* < 0.0001), VA showed a mean difference of 0.25 z-score (95% CI 0.06–0.43, *p* = 0.011), and KCO a mean difference of 0.35 z-score (0.35 (0.12–0.58), *p* = 0.0053). Lastly, 3/24 (13%) of the smaller twins had DLCO z-scores below the LLN versus 1/24 (4%) of the larger twins, with a risk ratio of 3.0 (95% CI 0.6–14.9; Fig. 2).

For lung volumes using helium dilution method, the absolute TLC in litres was lower for the smaller twin (3.32 (SD 0.89) versus 3.56 (SD 0.95)) as opposed to the larger twin.

A subgroup analysis of all outcomes in the twin pairs excluding the BPD cases (36/39 twin pairs, Table A1), showed that all differences between the smaller and larger twin found for the group as a whole persisted. In this subgroup of twins without BPD, asthma medication use was reported by 11% (4/36) of the smaller twins and 8% (3/36) of the larger twins.

## Discussion

In this study, we investigated the effect of FGR on lung function during childhood, using a unique study model of identical twins. Our data shows that FGR has a persistent negative effect on lung development and thereby lung function during childhood, independently of confounding factors such as genetic, obstetric, maternal, and postnatal environmental influences. Within-pair analysis revealed that the smaller twin presented with lower lung volumes as quantified by lower FEV_1_ and FVC z-scores, indicating restricted lung growth but with considerable uncertainty regarding airflow obstruction compared to the larger twin. Furthermore, this analysis showed that FGR in discordant twins is associated with a lower diffusion capacity of the lungs, even when taking the reduced lung volume into account.

Our findings add to the growing body of evidence that adverse fetal growth conditions have persistent negative consequences for lung development and thereby lung function during childhood.^4^, ^5^, ^6^ In a large study (n = 5.770) by Kotecha et al. it was found that in term born children, FGR was associated with significantly lower FEV_1_ and FVC z-scores at the age of 8–9 years.^5^ A meta-analysis performed by Saad et al. showed a reduction in FVC in adults born with a lower birthweight as well, whereas the association between birthweight and the FEV_1_/FVC ratio was weaker. However, the described findings in singletons can also be influenced by alternative confounding factors, including genetic factors, maternal smoking, prematurity, and postnatal environmental factors.

Current literature on lung function after sFGR in twins, in which these confounding factors are eliminated, is scarce.^8^, ^9^, ^10^, ^11^ A recent study by Salem et al. conducted a within-pair comparison of spirometry and lung MRI in MC twins with sFGR. They found that low birthweight was associated with lower FEV_1_ and FVC z-scores, which is consistent with our findings, and ventilation inhomogeneity of acinar airways, indicating reduced large and small airway function during adolescence.^8^ However, these results may have been influenced by the relatively small sample size (n = 20) and the inclusion of twin pairs with other MC twin pregnancy complications (in particular TTTS and TAPS). Moreover, two small studies by Nikolajev et al. investigated the association between FGR and lung function during childhood in a longitudinal discordant twin cohort and demonstrated contradicting results within these studies, with decreased lung function and increased bronchial reactivity but no effect on lung volumes.^10^^,^^11^ Yet, the generalizability of these results is limited, given the absence of z-scores and a lack of distinction in either zygosity or chorionicity.

The reported use of asthma medication in our study population was slightly higher in the smaller twin compared to the larger twin, namely 13% versus 8%. Yet, our analysis showed uncertainty regarding disparities in obstructive lung problems. In a previous twin study, Örtqvist et al. identified no associations between fetal growth and FEV_1_/FVC ratio either pre- or post-bronchodilator, and no indication that asthma serves as mediator between fetal growth and lung function.^9^ Consequently, they stated that FGR was primarily associated with a restrictive lung function pattern.

FGR can affect lung structure and function through multiple pathophysiological mechanisms in utero, including reduced nutrient supply, chronic hypoxia, chronic stress leading to hypercortisolaemia and epigenetic changes.^1^^,^^2^ Chronic hypoxia as caused by FGR is thought to impair pulmonary angiogenesis, by way of reactive oxygen species and alteration in amount of growth factors, which can affect pulmonary alveolarization.^25^^,^^26^ The smaller number of enlarged alveoli and a thickened blood-gas barrier can result in impaired gas exchange of the lung.^1^^,^^27^ Our study revealed that the diffusion capacity of the lung was significantly decreased for the smaller twin as opposed to the larger twin, with almost doubled odds for having a DLCO below the LLN. Specifically, the smaller twin has demonstrated lower KCO z-scores, which reflect the diffusion capacity per unit through the quality of alveolar-capillary gas exchange, while taking into account the VA. Thus, impaired lung development after FGR may have a shared pathophysiology with BPD, as FGR is a well-known risk factor for BPD.^28^ A subgroup analysis of all outcomes in the twin pairs excluding the BPD cases showed that all significant differences persisted, supporting the hypothesis that FGR, independently of BPD, influences lung function during childhood.

Given the multifactorial pathophysiology, it remains challenging to determine the precise mechanism underlying the association between FGR and reduced lung function during childhood. Although the lung function deficit was modest in most children, there is a clear association that warrants consideration in clinical practice. Namely, as subsequent lung function decline starts in early adulthood, children with lower lung volumes will reach clinically important lung function deficits earlier in life, making them more susceptible for respiratory morbidities. Therefore, counselling parents on additional risk factors for lung function decline, such as smoking, is essential, as early intervention can be crucial in preventing respiratory morbidities in later life.^3^ In addition, it may be beneficial to screen these children by using spirometry, especially in those with multiple risk factors including concomitant prematurity or BPD.

This study has certain limitations that should be considered when interpreting the results. Firstly, we have only included live-born twin pairs potentially leading to underrepresentation of cases with more severe outcomes. In addition, among the three excluded twin pairs due to spirometry of insufficient quality, two smaller twins had BPD, which may have led to an underrepresentation of twins with a high risk of lung function impairment. Yet, referral bias may have enlarged the proportion of twins at risk of severe postnatal course, as our centre is the national referral centre for complicated MC twin pregnancies in the Netherlands. Secondly, the retrospective review of neonatal respiratory morbidity over a large time span may have introduced historical bias as neonatal care has evolved over time. However, this bias is limited by our within-pair comparisons, both twins received the same neonatal care at the time. Additionally, the addition of CO-diffusion and helium dilution method later in the study led to relatively smaller sample sizes for these specific tests. Furthermore, as we did not use bronchodilators during spirometry, reversibility was not assessed. Lastly, given the distinct etiological mechanisms underlying sFGR in twins and FGR in singletons, our twin design may not serve as a full surrogate for the latter. Where FGR in singletons is multifactorial in origin, primarily including placental insufficiency, sFGR is presumed to be caused by unequal placental sharing.^29^^,^^30^ Therefore, caution must be taken when generalizing our results. To further broaden our knowledge, more conclusive evidence is needed on achieving maximum lung capacity after FGR, for example, by re-inviting these twins around the age of 18 years and thereby obtaining longitudinal data on lung function trajectories. Moreover, in future prospective studies, it would be valuable to collect data on differences in peripheral oxygen saturation (SpO_2_) and fraction of inspired oxygen (FiO_2_) during the neonatal period, as well as respiratory health throughout childhood, including pharmacological therapy and other medical interventions would add valuable information. In addition, the inclusion of a control group of appropriately-grown matched singletons would improve the generalizability of our findings in singletons with FGR. Nevertheless, we present an extensive overview of lung function throughout childhood in a relatively large cohort of identical twins with sFGR. Where previous studies solely reported data on spirometry, we have included lung volumes using helium dilution and diffusion capacity. By using an identical twin model, we eliminated fundamental confounders that influence lung function throughout childhood.

In conclusion, we provide a comprehensive analysis of lung function during childhood after FGR, including lung volumes and diffusion capacity, using a unique model with growth discordant identical twins, thereby controlling for genetic, obstetric, maternal, and postnatal environmental influences on childhood lung function trajectories. We show that the smaller twin had a reduction in static lung volume with an equivalent decrease in dynamic lung function, and a reduction in diffusion capacity compared to the larger twin. Although the lung function deficit was modest in most children, having reduced reserve capacity in the smaller twin can contribute to an increase in respiratory morbidities later in life. Therefore, screening in those with concomitant risk factors and timely counselling of additional hits on lung function is warranted in those born after FGR.

## Contributors

Spekman, de Vreede, Roest, van Klink, Lopriore and Groene were responsible for the concept and design of the study. Spekman, Israëls, de Vreede, Los, Lopriore and Groene analysed and interpreted the data. Spekman, van Zwet and Groene did the statistical analysis. Spekman and Groene drafted the manuscript. All authors critically revised the manuscript. Spekman, Israëls, Lopriore and Groene accessed and verified the data. All authors had full access to all the data in the study and had final responsibility for the decision to submit for publication.

## Data sharing statement

Individual participant data (including data dictionaries) that underlie the results reported in this article, after de-identification, and the study protocol will be available beginning 3 months and ending 10 years after publication. Data will be shared with researchers who provide a methodologically sound proposal and whose proposed use of the data has been approved by an independent review committee identified for this purpose and the medical ethical committee of the Leiden University Medical Centre, to achieve aims in the approved proposal. Proposals should be directed to Lemon_KJC@lumc.nl. To gain access, data requestors will need to sign a data access agreement.

## Declaration of interests

The authors declare no competing interests.

## Appendix A Supplementary data

Table A1
