# Supplementary material for: Reduced lung function during childhood in identical twins with discordant fetal growth: a cohort study
Source: eClinicalMedicine. 2024 Apr 10;72:102600. doi: 10.1016/j.eclinm.2024.102600 (PMC11019090; doi:10.1016/j.eclinm.2024.102600)
Supplement: Table A1 [file mmc1.pdf]

Table A1. Subgroup analysis of within-pair comparison of spirometry, CO-diffusion, and helium dilution outcomes between the smaller and larger twin in MC twins with sFGR excluding the BPD cases.

	Smaller twin (n=36)	Larger twin (n=36)	*p*-value	Mean difference (95%CI)
Age at follow-up (years)	11·7 (3·4)		··	··
Height at follow-up (cm)	149·6 (20·7)	152·2 (20·9)	<0·0001	2·6 (1·6—3·6)
**Spirometry (n=35)[a]**				
FEV_1 (L)	2·35 (1·03)	2·58 (1·07)	··	0·23 (0·12—0·34)
z-score	-0·86 (0·98)	-0·35 (1·13)	0·0046	0·51 (0·17—0·85)
FVC (L)	2·82 (1·25)	3·09 (1·37)	··	0·27 (0·18—0·36)
z-score	-0·50 (0·81)	-0·01 (1·06)	0·00022	0·48 (0·25—0·72)
FEV_1/FVC	83·56 (7·53)	84·20 (6·86)	0·63	0·64 (-2·08—3·37)
VC MAX (L)	2·87 (1·22)	3·14 (1·34)	··	0·27 (0·18—0·36)
z-score	-0·41 (0·92)	0·00 (1·09)	0·00014	0·41 (0·22—0·61)
FEV_1/VC MAX	82·94 (8·18)	83·58 (6·81)	0·66	0·64 (-2·29—3·56)
**CO-diffusion method (n=21)**				
DLCO (mmol/(min*kPa))	5·47 (1·53)	6·18 (1·74)	··	0·71 (0·40—1·03)
z-score	-0·32 (0·79)	0·18 (0·74)	0·0011	0·50 (0·22—0·77)
VA (L)	3·09 (0·95)	3·35 (1·03)	··	0·25 (0·12—0·39)
z-score	-0·36 (0·94)	-0·12 (0·92)	0·020	0·25 (0·04—0·45)
KCO (mmol/(min*kPa))	1·80 (0·24)	1·88 (0·25)	··	0·07 (0·01—0·14)
z-score	-0·07 (0·66)	0·24 (0·70)	0·017	0·32 (0·06—0·57)
**Helium dilution method (n=18)**				
RV (L)	0·88 (0·29)	0·86 (0·29)	··	-0·02 (-0·13—0·09)
z-score	0·17 (0·45)	0·02 (0·52)	0·36	-0·14 (-0·47—0·18)
TLC (L)	3·33 (0·94)	3·53 (0·98)	··	0·20 (0·05—0·34)
z-score	-0·29 (0·99)	-0·14 (0·76)	0·27	0·14 (-0·12—0·41)
% predicted	96·7 (14·2)	98·4 (10·3)	0·34	1·7 (-2·0—5·4)
RV/TLC	26·57 (5·85)	24·58 (7·49)	0·24	-1·98 (-5·42—1·46)

Outcomes are presented as mean (SD) and as mean difference (95%CI). All analysis were performed using the paired t-test. The within-pair difference is calculated as: outcome larger twin – outcome smaller twin. Cm: centimetres, FEV_1: forced expiratory volume in one second, L: litres, FVC: forced vital capacity, VC MAX: maximum vital capacity, CO: carbon monoxide, DLCO: diffusing capacity of the lung for carbon monoxide, mmol: millimoles, min: minutes, kPa: kilopascal, VA: alveolar volume, L: litres, KCO: carbon monoxide transfer coefficient, RV: residual volume, L: litres, TLC: total lung capacity. [a]Not all spirometry outcomes were of sufficient quality in all of the 36 twin pairs, resulting in outcome measures available in either 34 (FEV_1/VC MAX) or 35 (all other outcomes) twin pairs.
